# P-2104. Revolutionizing HIV Care: The Impact of Telemedicine on Achieving Viral Load Suppression in Outpatient Settings

**DOI:** 10.1093/ofid/ofaf695.2268

**Published:** 2026-01-11

**Authors:** Aung Naing, Suna Horie, Aleksandra Bulaeva, Nicholas Schouten, Fang Fang, Kripa Ahuja, Max Prokopy, Catherine Derber

**Affiliations:** Eastern Virginia Medical School at ODU, Norfolk, Virginia; EVMS at ODU, Norfolk, Virginia; EVMS at ODU, Norfolk, Virginia; EVMS at ODU, Norfolk, Virginia; Virginia Health Sciences at ODU, Norfolk, Virginia; EVMS at ODU, Norfolk, Virginia; University of Virginia, Charlottesville, Virginia; Eastern Virginia at ODU, Norfolk, Virginia

## Abstract

**Background:**

Consistent access to care is crucial for optimizing viral load suppression in patients with HIV (PWH). The COVID-19 pandemic offered a unique opportunity to evaluate the role of telemedicine in achieving and maintaining an undetectable viral load in PWH. We examined the impact of telemedicine on HIV viral load suppression and retention in care relative to in-person visits.

**Methods:**

A single-center retrospective study was conducted among PWH aged 18-89 years old having at least one appointment in each Pre-COVID (3/1/2018-2/28/2020) and COVID (3/1/20-2/28/23) period. Only in-person visits were available during the pre-COVID time period, but both in-person and telemedicine visits were available during the COVID period. Deceased and pregnant patients during the study period were excluded. Generalized linear mixed effect model was used to examine the associations between telemedicine and outcomes among PWH considering patients’ characteristics across the periods.

**Results:**

A total of 368 patients were included in the analysis. There was no difference in attendance rates to scheduled appointments and rates of HIV viral load suppression between pre-COVID and COVID periods. Patients were more likely to have HIV viral load testing every 6 months during the pre-COVID period (62.77% vs 33.42%, p< 0.001). Multivariable analysis showed that telemedicine had no impact on either of the outcomes, while being in the COVID period remained a negative impact on HIV viral load testing (OR=0.21, 95% CI=0.11-0.37, p< 0.001). Having a medical comorbidity, mental illness diagnosis or substance use disorder did not affect appointment adherence, viral load testing or viral suppression.

**Conclusion:**

Telemedicine complements in-person care by improving access to HIV care without compromising quality. DHHS guidelines recommend HIV viral load testing every 6 months, but patients in our study were less likely to meet this goal during COVID-19. Despite this telemedicine did not affect appointment adherence or viral suppression rates. Limitations to this study include the small sample size andsingle-center study. Further research could explore ways to promote access for PWH using telemedicine and determine the optimal lab testing frequency for individuals with suppressed HIV on stable antiretroviral regimens.

**Disclosures:**

Catherine Derber, MD, Kaplan Power of Knowledge (they received an educational grant through ViiV): Honoraria

